# Comparison of hemorrhoidectomy by LigaSure with conventional Milligan Morgan’s hemorrhoidectomy

**DOI:** 10.12669/pjms.323.9976

**Published:** 2016

**Authors:** Nighat Bakhtiar, Foad Ali Moosa, Farhat Jaleel, Naeem Akhtar Qureshi, Masood Jawaid

**Affiliations:** 1 Dr. Nighat Bakhtiar, MBBS. Post Graduate General Surgery Fellowship trainee, Department of Surgery, Dow International Medical College/ Dow University Hospital, Dow University of Health Sciences, Karachi, Pakistan. Department of Surgery, Dow International Medical College/ Dow University Hospital, Dow University of Health Sciences, Karachi, Pakistan; 2 Prof. Foad Ali Moosa, MBBS, FRCS. Professor & Head, Department of Surgery, Dow International Medical College/ Dow University Hospital, Dow University of Health Sciences, Karachi, Pakistan; 3 Dr. Farhat Jaleel, MBBS, FCPS (General Surgery). Associate Professor, Department of Surgery, Dow International Medical College/ Dow University Hospital, Dow University of Health Sciences, Karachi, Pakistan; 4 Dr. Naeem Akhtar Qureshi, MBBS, FCPS (General Surgery). Assistant Professor, Department of Surgery, Dow International Medical College/ Dow University Hospital, Dow University of Health Sciences, Karachi, Pakistan; 5 Dr. Masood Jawaid, MBBS, MCPS, MRCS, FCPS (General Surgery), MHPE. Visiting Faculty, University of Health Sciences, Lahore, Pakistan

**Keywords:** Heamorrhoidectomy, LigaSure, Milligan Morgan, Efficacy

## Abstract

**Objective:**

To compare the efficacy of haemorrhoidectomy done by using LigaSure with conventional Milligan Morgan haemorrhoidectomy.

**Methods:**

This randomized controlled trial was done at Department of Surgery Dow University Hospital Karachi during January 2013 to September 2015. A total of 55 patients were included in the study. Patients were randomly allocated to group A (Haemorrhoidectomy by Ligasure) and group B (Milligan Morgan Haemorrhoiectomy). Efficacies of both procedures were compared by operative time, Blood loss, wound healing, and pain score on immediate, 1st and 7^th^ post operative day.

**Results:**

Out of total 55 patients 23 were male and 32 were females. The most common group of age involved was between 40 – 60 years. Third degree Heamorrhoids were present in 37 (67.3%) of patients while remaining 18 (32.7%) had fourth degree Heamorrhoids. Group A included 29 cases while Group B included 26 cases. The mean operating time of Group A was 52.5 with standard deviation of 11.9 while it was 36.6± 9.8 in the other group. The mean blood loss in group A was 51.92 with standard deviation of 15.68 while it was 70.34±25.59 in group B. Overall pain score was less in those patients who underwent Heamorrhoidectomy by Ligasure method.

**Conclusion:**

The efficacy of Heamorrhoidectomy by Ligasure is better than the traditional Milligan Morgan Heamorrhoidectomy but we need more clinical trials with large sample size and long term follow ups.

## INTRODUCTION

Hemorrhoids, a varicose condition is one of the commonest illnesses which causes per rectal bleeding.^1^ The main effective and ultimate treatment for 3^rd^ or 4^th^ degree haemorrhoids is Haemorrhoidectomy.^2^ Numerous other procedures have also been practiced, varying from open or closed sharp excision, laser therapy, ultrasonic scalpel dissection to stapled Hemorrhoidectomy.^3^-^6^ Even though Haemorrhoidectomy is thought to be a small procedure but the complications and the postoperative recovery are very painful to the patient and maybe that’s the reason why patients consider haemorrhoidectomy as the last option of treatment. Patients as well as surgeons do not like Haemorrhoidectomy because as it is painful for the patient in the same way it is considered to be a difficult procedure among many surgeons.

Traditional Milligan Morgan haemorrhoidectomy is the open surgical procedure in which the haemorrhoid pedicle is ligated by a transfixing suture which may lead to some postoperative complications mostly pain, bleeding and wound infection which ultimately cause prolonged stay in hospital. A number of surgeons believe that by avoiding vascular pedicle ligation the chances of secondary bleeding can be decreased. The reason behind this is belief that it may lead to ischaemia and necrosis at the region where these sutures are applied it may also integrate the sphincter muscle and consequently causes acute postoperative pain, wound infection and bleeding. Additionally if the sutures are applied deeply they can also cause firm circular scarring at the anus later on. Therefore, many authors have said that we do not transfix vascular pedicles of haemorrhoids, but we seal them by LigaSure.^7^

The LigaSure vessel sealing system is a bipolar electro-thermal device which seals the blood vessels by a calculated arrangement of pressure and radio frequency.^8^ Another new and advanced technique called Stapled haemorrrhoidectomy is also practiced by few skilled surgeons but its results are not still conclusive.^9^ For Ligasure there is no need of special skilled persons as needed for stapled haemorrhoidectomy because the procedure done by LigaSure is very identical to that used in conventional haemorrhoidectomy.

Little research has been done about the outcome of Ligasure, but it has been proven as the simple and safe surgical treatment of haemorrhoids in terms of operative time, postoperative pain and wound infection as compared to conventional haemorrhoidectomy by some experimental studies.^10^,^11^ One study done in India showed that it is safe and effective and has less blood loss, postoperative pain and complications compared to conventional hemorrhoidectomy,^12^ but no similar research has been done in Pakistan yet.

The aim of this study was to verify that haemorrhoidectomy done by Ligasure tissue sealing system is an effective alternate procedure as compared to the usual traditional haemorrhoidectomy. The search for a better new procedure of hemorrhoidectomy for the patients in our country made us to use an electro thermal device which was used to seal vessels in abdominal and thyroid surgery formerly and as its effectiveness has not been measured in Pakistan yet. We used this apparatus to perform a procedure that effectively achieves a suture less hemorrhoidectomy which is less painful and less distressing for the patient with decreased frequency of complications and as well as it is easy to use and less time consuming for the surgeon. The results of this study helped us to confirm that the LigaSure hemorrhoidectomy is a useful new method for treatment for patients diagnosed with haemorrhoids and it also helped us in verifying the effectiveness of LigaSure hemorrhoidectomy as a new alternate and effective procedure in Pakistan.

## METHODS

This randomized controlled trial was done at Department of Surgery Dow University Hospital Karachi during January 2013 to September 2015, after the approval of Research and training monitoring cell of CPSP. A total of 55 patients were included in the study. This sample size has been calculated using the formula, based on hypothesis test for two population proportions (one sided test). All patients with ages between 18 to 70 years of both genders with third and fourth degree Haemorrhoids admitted in department of general surgery of Dow University Hospital planned for Surgery, giving informed consent for haemorrhoidectomy done by using LigaSure and conventional Milligan Morgan haemorrhoidectomy were included in the study. Patients who were undergoing a combined procedure for fissures or fistulae or those having other conditions like thrombosed haemorrhoids, inflammatory bowel diseases and immune deficiency due to AIDS were not included. The patients who were previously operated upon for haemorrhoids were also excluded.

Patients were randomly allocated to group A (Haemorrhoidectomy by Ligasure) and group B (Milligan Morgan Haemorrhoiectomy) by using the random allocation software version 1.0.0 Patients were blinded to the type of surgery performed. A standardized spinal or general anesthesia was used. The procedure was carried out with the patient in lithotomy position and a minor reverse Trendelenberg angle. The primary steps in both surgeries were same and consisted of Examination under anesthesia, delivery of hemorrhoids by artery forceps, one applied at the muco cutaneous junction of hemorrhoid, the other at the apex and a skin incision at the base of hemorrhoids and separation of hemorrhoid tissue from the internal sphincter fibers by monopolar diathermy or scissors.

After this in the Millagan Morgan’s procedure the hemorrhoid pedicle was transfixed with 0 number Vicryl suture. In the Ligasure group the jaws of the handset were applied on the pedicle and the instrument activated by the foot paddle. A digitally managed feedback circuit automatically stopped the flow of energy when coagulation of the vessels and mucosa was achieved. No sutures were applied as the Ligasure device also achieved mucosal fusion. Anal canal packing was not usually done except when there was any doubt about securing heamostasis.

The operative time was recorded by an operating theatre nurse. Blood loss was recorded as the number of soaked gauzes. Soakage of one 4×4 gauze piece was considered as 30 ml blood loss. A standard medication package was given to all patients postoperatively. All patients were advised injectble antibiotics Ciproxin 400mg twice daily and Metronidazole 500mg thrice daily with inject able ketocrolac 30 mg thrice daily during intial 24 hours post op period. All patients were advised to take Isphaghoul husk two table spoon full twice a day for two weeks to aid in defecation after the operation. All patients were prescribed Metronidazole 400mg thrice daily and Ciproxin 500mg twice daily. Paracetamol two tablets three times daily and Diclofenac 50 mg as per required orally were advised to all patients for postoperative analgesia.

The patients were discharged on the first postoperative day unless otherwise clinically indicated. All patients were asked to clean the wound doing sits bath twice daily. Patients were then followed up in the clinic 1^st^, 2nd, 3^rd^ and 4^th^ week after discharge. Patients were taught with an 11-point visual analogue pain score from zero to ten. The patients were asked to record at home before bed-time their maximum pain score for the day. Wound healing assessment was done at planned appointments. The grade of the wound was assessed by presence of granulation tissue on 14^th^ post operative day. This was done in OPD by parting the buttocks and inspecting the wound.

Statistical analysis was performed with SPSS software version 17. Independent sample T- test was applied to compare the operative time, blood loss and post operative pain in both groups. Post stratification Independent Sample T- test was applied; value ≤ 0.05 will be taken as significant.

## RESULTS

**Fig.1:**
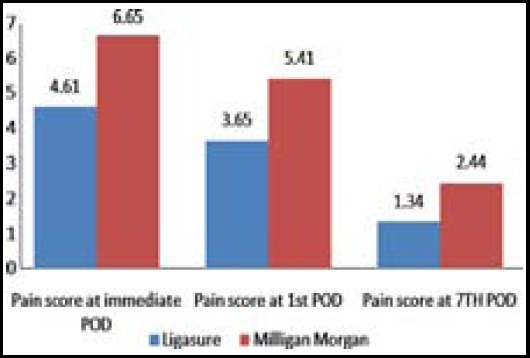
Comparison of mean pain score of both groups at immediate, 1^st^ and 7^th^ POD.

Out of total 55 patients 23 were male and 32 were female. The most common group of age involved was between 40 – 60 years. Third degree Heamorrhoids were present in 37 (67.3%) of patients while remaining 18 (32.7%) had fourth degree Heamorrhoids. Group A included 29 cases in which 20 were having 3^rd^ degree heamorrhoids while Group B included 26 cases in which 17 were having 3^rd^ degree heamorrhoids. The mean operating time of Group A was 52.5 minutes with standard deviation of 11.9 while it was 36.6± 9.8 in the other group. The mean blood loss in group A was 51.92ml with standard deviation of 15.68 while it was 70.34±25.59 in group B. Overall pain was less in those patients who underwent Heamorrhoidectomy by Ligasure method shown in Table-I and Fig.1. The wound healing which was assessed by appearance of granulation tissue on 14^th^ post operative day was observed in 24 (n=29) patients of group A but granulation tissue appeared only in 16 patients (n=26) in group B.

**Table-I:** Comparison of operative outcomes in patients undergoing Ligasure and Milligan Morgan’s hemorrhoidectomy.

	*Ligasure Group A (n=29)*	*Milligan Morgan Group B (n=26)*	*P value*
Males	09	14	
3RD Degree	20	17	
4th Degree	09	09	
Mean Operative time(minutes)	36.6(9.8)	52.5(11.9)	0.001
Blood loss(in ml)	51.92(15.68)	70.34(25.59)	0.003
Pain score at immediate POD	4.61(0.80)	6.65(0.97)	0.001
Pain score at 1st POD	3.65(0.79)	5.41(0.68)	0.001
Pain score at 7TH POD	1.34(0.56)	2.44(0.68)	0.001
Wound Healing (Appearance of granulation tissue on 14th POD)	24	16	

**Fig.2:**
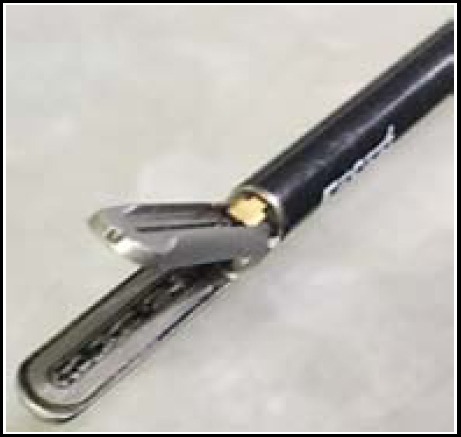
The Ligasure Device

## DISCUSSION

It is always observed that innovative methods can give a faster and safer technique for any surgical procedure. Considering the major complaints during this difficult procedure Heamorrhoidectomy by LigaSure is a newly designed technique which helps in decreasing the complications of this surgical procedure and it is compared to other traditional heamorrhoidectomy procedures in many published randomized trials.^13^,^14^

The major decrease in post operative pain from immediate post operative day to 7^th^ post operative day in patients undergoing the LigaSure technique, i.e. from 4.6 to 1.34 compared to 6.65 to 2.44 in the other group observed, supports the idea that the new technique of using Ligasure for Heamorrhoidectomy causes decreased postoperative pain. These results are in comparable with another study done in India which reported the post operative pain on immediate post operative day as 4.1±0.8 which decreased to 1.2±0.2 on 7^th^ post operative day.^15^

Similarly the mean operative time in this study was 36.6 in Ligasure group which was less as compared to the other group having 52.5 minutes comparable to other studies which also showed less time consumption of Ligasure procedure.^15^ The bleeding during surgery was also less compared to the Milligan Morgan method because of the reason that it effectively achieve heamostasis by complete coagulation of the vessel that’s why I is also called ‘vessel sealing system’. The flow of energy is automatically stopped by a computer controlled feedback System when complete coagulation is achieved.

The major limitations for this study was the small sample size and short follow up of the patients as compared to previous studies^4^ which will be overcome in the next trial with long follow ups and observation of late complications. The basic disadvantage with the LigaSure technique in our locality is its expensive cost but this disadvantage has been noted with all new techniques. The charges of the electrode per patient increased to approximately 5000 Rupees per patient which signify increase in the cost of the procedure. On the other hand, if we see the short operative time ultimately saves per minute charges of the patient in this less time consuming procedure. Therefore, the total cost-saving per procedure is ultimately equal in both techniques either open or Ligasure Heamorrhoidectomy. Regardless of all these disadvantages we suppose that this study is valuable because randomization was equally matched in the two groups in terms of age, gender and follow up of the patients. Another reason for using LigaSureTM for hemorrhoidectomy is the idea that this vessel sealing system considerably decreases the thermal spread in comparison to the diathermy instrument. This device LigaSure system specifically limits thermal spread to adjacent 2 mm tissue. So generally less amount of thermal injury at the surgical site leads to decrease postoperative pain.^16^

Even though encouraging preliminary results of the studies are available about this new surgical technique with less number of complications but we need to do more prospective trials comparing the two groups of Ligasure to the traditional one with large sample size and long term follow ups for recurrence to conclude its definite good efficacy, so that it will become a good option of treatment for third and fourth degree heamorrhoids.

In future this procedure can be done in local anesthesia as some of the researchers have already done work on this to decrease the cost and increase the efficacy of the technique but this again needs further clinical trials in our locality.

## CONCLUSION

The efficacy of Heamorrhoidectomy by Ligasure is better than the traditional Milligan Morgan Heamorrhoidectomy but we need more clinical trials with large sample size and long term follow ups.
